# Responses of tree growth, leaf area and physiology to pavement in *Ginkgo biloba* and *Platanus orientalis*

**DOI:** 10.3389/fpls.2022.1003266

**Published:** 2022-12-01

**Authors:** Bowen Cui, Xuming Wang, Yuebo Su, Cheng Gong, Danhong Zhang, Zhiyun Ouyang, Xiaoke Wang

**Affiliations:** ^1^ State Key Laboratory of Urban and Regional Ecology, Research Center for Eco-Environmental Sciences, Chinese Academy of Sciences, Beijing, China; ^2^ College of Resources and Environment, University of Chinese Academy of Sciences, Beijing, China; ^3^ Key Laboratory for Subtropical Mountain Ecology (Ministry of Science and Technology and Fujian Province Funded), College of Geographical Sciences, Fujian Normal University, Fuzhou, China; ^4^ Shenzhen Academy of Environmental Sciences, Shenzhen, China; ^5^ Beijing Urban Ecosystem Research Station, Chinese Academy of Sciences, Beijing, China

**Keywords:** urban trees, pavement, tree growth, leaf morphology and physiology, carbon isotopes

## Abstract

Trees growing on paved lands endure many environmental stresses in the urban environment. However, the morphological and physiological mechanisms underlying tree adaptation to pavement in the field are less known. In this study, we investigated 40 sites where *Ginkgo biloba* and *Platanus orientalis* grow on adjacent pairs of paved and vegetated plots in parks and roadsides in Beijing, China. Relative to the vegetated land, the mean increments in the diameter at breast height and height in the paved land were significantly decreased by 44.5% and 31.9% for *G. biloba* and 31.7% and 60.1% for *P. orientalis*, respectively. These decreases are related to both the decrease in assimilation products due to the reductions in leaf area, leaf total nitrogen content, and chlorophyll content and the increase in energy cost due to the synthesis of more soluble sugar and proline for mitigating stress. The increase in leaf soluble sugar content, proline content, and δ^13^C indicated that trees could adapt to the paved land through the regulation of osmotic balance and the enhancement of water-use efficiency. Piecewise structural equation models showed that trees growing on the paved land are stressed by compounding impacts of the leaf morphological and physiological changes. Therefore, it is critical to explore the complex response of plant morphological and physiological traits to the pavement-induced stress for improving tree health in urban greening.

## Introduction

Urban trees could provide multiple ecosystem services that are significantly beneficial to residents, such as improving air quality (Nowak et al., 2018), reducing noise pollution (Margaritis and Kang, 2017), increasing carbon sequestration (Nowak and Crane, 2002), and cooling air and surfaces (Armson et al., 2012) through shading effects and evaporative processes (Upreti et al., 2017; Wang et al., 2019). In recent years, urban tree planting programs have been carried on in many cities (Koesera et al., 2014), such as New York’s Million Trees initiative (City of New York, 2011) and Beijing’s Million Mu (Chinese unit of land area = 1/15 ha) Tree Planting initiative, to maximize and sustain ecological services (Pincetl et al., 2013). Because of the shortage of land available for tree planting in the urban context, paved lands such as roadside and public square have been deployed widely for planting more trees. However, the paved land does not favor plant growth because it alters soil microenvironments through increased soil surface and rhizospheric temperature (Arnfield, 2003; Tang et al., 2011), decreased rainwater infiltration (Lee and Heaney, 2003), aggravated soil compaction (Philip and Azlin, 2005), inhibited soil–air gas exchange (Feng et al., 2002; Balakina et al., 2005), reduced nutrient availability (Zhao et al., 2012), and changed energy and water balances (Morgenroth and Buchan, 2009). In general, the urban paved land is compacted with road rollers for smoothing. The compacted substrate could restrict plant growth by narrowing the soil pore spaces through which root tips extend and young roots expand radially (Grabosky et al., 2009). When compacted soils were encountered as they grow beyond the planting pits, the tree root growth was hindered due to the reduced soil porosity and water infiltration (Gregory et al., 2006; Pitt et al., 2008). Trees growing on the paved land have to endure a number of stresses [e.g., drought, heat, soil compaction, and pollution (Chen et al., 2017)] and disturbances [e.g., infrastructure installations and maintenance work, vandalism, and car accidents (Mullaney et al., 2015b)]. So, trees growing on the paved land often have high mortality rates, short average life spans, and low growth (Day and Amateis, 2011; Sand et al., 2018). Mueller and Day (2005) reported that *Nerium oleander* saplings planted in open lawn sites have better growth than those in sites surrounded by an impervious pavement. Chen et al. (2017) discovered that the pavement-induced rise in surface temperature decreased the survival and growth of the *Acer truncatum* sapling. Moser et al. (2017) found significant stem growth reductions in a highly paved public square compared with a contrasting more open and greener square. In contrast, some results showed that trees grow similarly or even greater under the paved soil than the non-paved one (Morgenroth, 2011; Morgenroth and Visser, 2011; Fini et al., 2017; Fini et al., 2022). Therefore, more investigations should be conducted to understand why trees respond inconsistently to the pavement.

The leaf, as the most important component that can transfer solar energy into chemical energy to support all life processes, is very plastic in response to environmental changes. The leaf can readily respond to stresses by alteration of its morphological and physiological traits. Mullaney et al. (2015b) explored the effects of different pavement types on the leaf total nitrogen (TN), stomatal conductance, and δ^13^C of *Melaleuca quinquenervia* at different growth stages, which were all correlated with photosynthetic capacity. The pavement induced a reduction in chlorophyll content and an increment in malondialdehyde (MDA) in *A. truncatum* leaves (Chen et al., 2016). However, little is known about how the leaf morphological characteristics adapt to the pavement, including leaf area and specific leaf area (SLA), both of which can be strongly affected by environmental stress (Parkhurst and Loucks, 1972; Poorter et al., 2009).

In this study, two species of common urban trees, one gymnosperm, *Ginkgo biloba*, and one angiosperm, *Platanus orientalis*, are studied because they are the most widely planted urban trees (Dmuchowski et al., 2019; Vrinceanu et al., 2021) and highly resistant to drought (Roloff et al., 2009) at sites that each has adjacent pairs of paved and vegetated lands. The environmental factors, tree growth, and leaf morphological and physiological traits are investigated *in situ*. The following three questions will be answered: 1) Are there differences in soil temperature, moisture, and nutrient between paved and vegetated lands? 2) What changes in leaf morphological and physiological traits occur to adapt to the paved land? 3) Do *G. biloba* and *P. orientalis* respond differently to the paved land?

## Materials and methods

### Study sites

This study was conducted in the urban area of Beijing (39°56′N, 116°20′E). It is the capital of China with more than 20 million residents and is characterized by a typical continental monsoon climate with distinct seasons. The mean annual precipitation and temperature are around 500 mm and 11°C–12°C, respectively. The soils are mixtures of original fluvo-aquic soil, fine sand, and clay and vary with site history and construction. The average saturated soil water content and hydraulic conductivity were 40.6% and 2.4 × 10^-4^ (cm·s^-1^), respectively (Xie et al., 1998).

**Figure 1:**
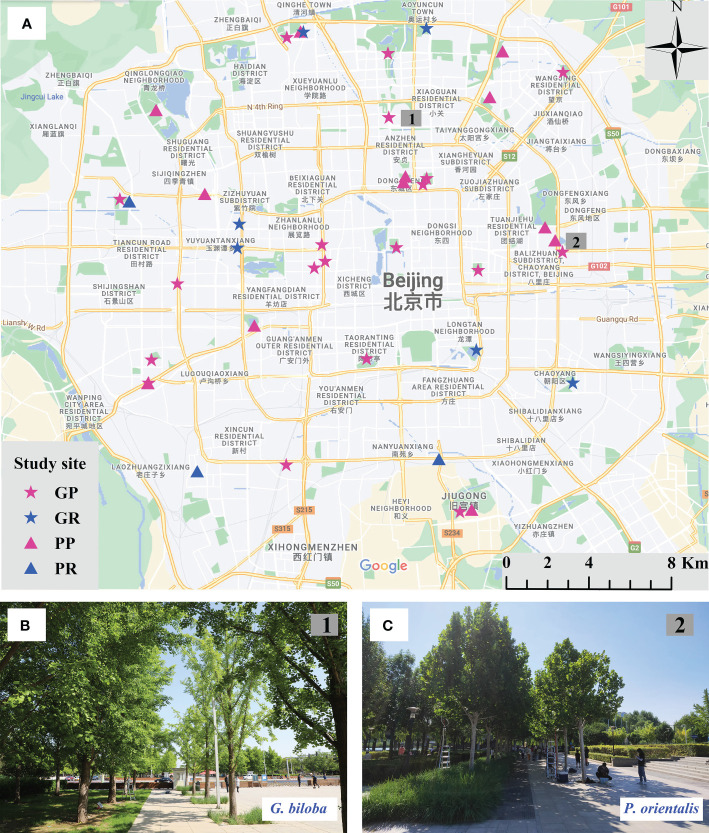
Location of the 40 study sites **(A)** for *Ginkgo biloba* at parks (GP) and at roadsides (GR) and for *Platanus orientalis* at parks (PP) and at roadsides (PR). Study sites of *G biloba* in Minzuyuan park **(B)** and *P. orientalis* in Honglingjin park **(C)**. At each study site, trees grow at adjacently paired plots: vegetated land and paved land.

**Figure 2:**
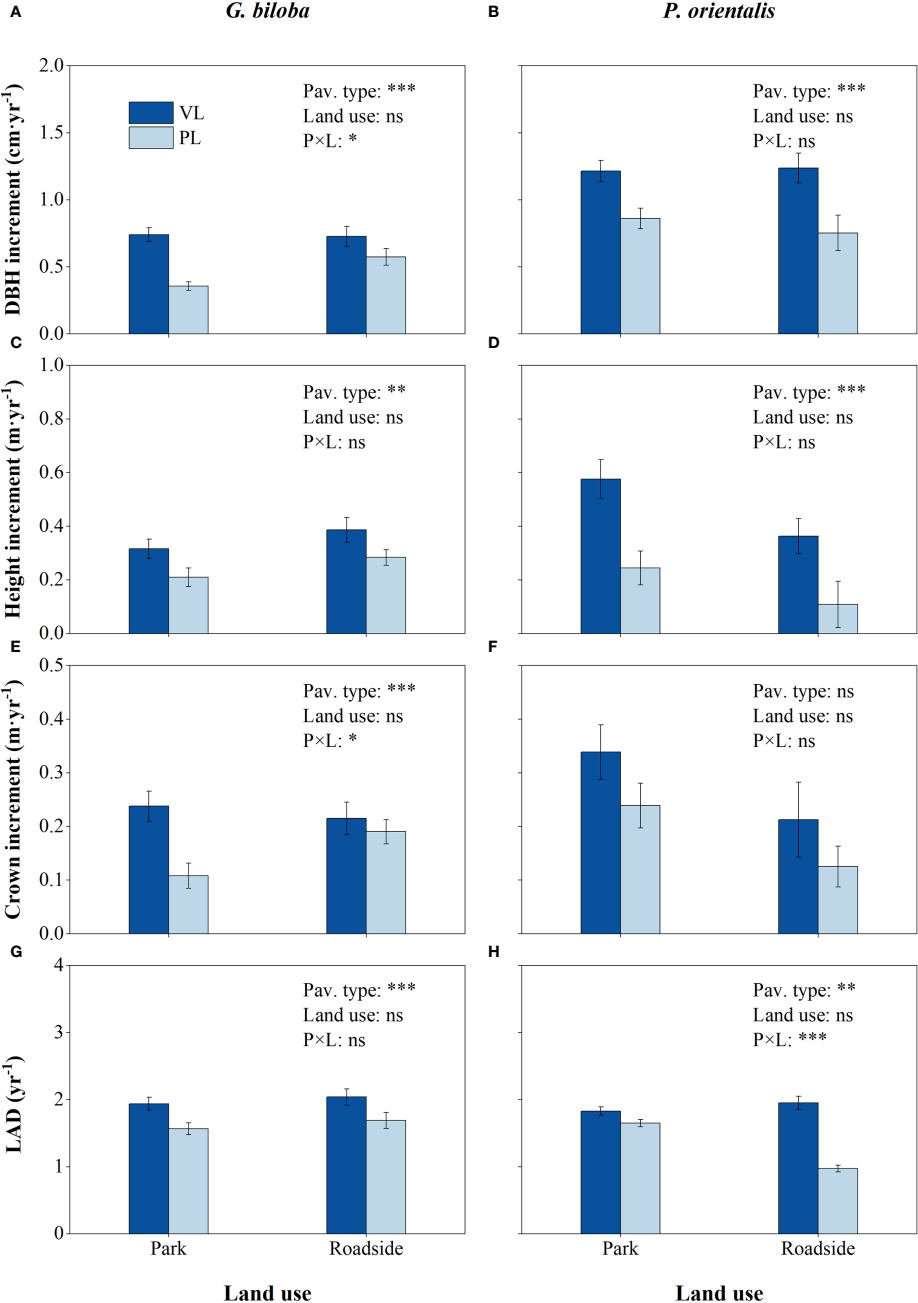
Results from the linear mixed-effects model (LMM) testing for the differences in the DBH increment **(A, B)**, height increment **(C, D)**, crown increment **(E, F)**, and leaf area density (LAD) **(G, H)** vs. the pavement type (VL, vegetated land; PL, paved land), land use (park and roadside), and their interactions. *, *P*< 0.05; **, *P*< 0.01; ***, *P*< 0.001; ns, not significant. Data represent mean ± SE.

In parks and roadsides within the 5th Ring Road of Beijing, spatially balanced study sites were selected (**Figure 1A**). A total of 40 sites were finally selected, of which 25 sites were for *G. biloba* that were located in 19 parks and six roadsides, and 15 sites were for *P. orientalis* that were located in 12 parks and three roadsides. At each site, one of two species of *G. biloba* and *P. orientalis* grows at adjacently paired plots: paved and vegetated lands (**Figures 1B, C**). Paved lands are mostly covered by marble or impervious brick and a small amount by pervious brick on which trees grow in pits usually in the size of 2 m × 2 m and 1 m × 1 m for park and roadside, respectively. Soils under the paved land were usually compacted for supporting the expected pavement loading. Vegetated lands are occupied by turf, shrub, or ruderal plants. Meanwhile, the trees at each site have the same age and are managed equally, and at each plot, there are more than three individuals of target trees. The tree ages varied from 20 to 40 years in different study sites for both *G. biloba* and *P. orientalis*, which were inferred from the size of the trees, including the diameter at breast height (DBH) and height, which are shown in **Figure 2**. Study sites where trees were pruned during the experiment were not counted in this study.

### Field measurements

On each pair of paved and vegetated plots, we measured DBH, height, crown radius, and leaf area density (LAD; total one-sided leaf area per unit volume) of 560 individuals of *G. biloba* and *P. orientalis* in both years of 2018 and 2021 at the interval of 3 years. DBH was measured with a steel dendrometer band at 1.3 m aboveground, except in the case of buttressed stems, which were measured above the buttress to avoid overestimation (Condit, 1998). Height was determined as the height from the root collar to the top of the highest living bud using a Nikon Forestry Pro (Nikon Vision, Tokyo, Japan) laser rangefinder to the accuracy of 0.1 m. Crown radius was the average distance from the center of the trunk to the farthest point below branch tips in four directions, north, south, east, and west, which was measured using a linear tape. LAD was measured by the LAI-2000 Plant Canopy Analyzer (Li-Cor, Inc., Lincoln, NE, USA).

At each pit where trees were measured, the surface temperature, soil temperature, and soil moisture were remeasured during each investigation. The surface temperature and soil temperature were measured using an infrared thermometer (Optris MS, Optris GmbH, Berlin, Germany) and soil thermometer (SYS-TP101, SYS, Liaoning, China). The soil moisture was measured using TDR300 (Spectrum Technologies Inc., Plainfield, IL, USA).

### Soil sampling and analysis

Soils were sampled at paired plots of 15 study sites (eight sites for *G. biloba* and seven sites for *P. orientalis*) in October 2021. In each plot, soil (0–20 cm) was collected at three randomly selected tree pits using a soil drill (4-cm diameter) and mixed as a composite soil sample. A total of 90 soil samples were collected. Soil samples were kept in a ziplock plastic bag. After transferring to the laboratory, soil samples were stored at -20°C for further analysis.

After removing litters, stones, and debris, the soil samples were homogenized by passing through a 10-mesh (2-mm) sieve and then air-dried at 22°C for 15 days. All samples were ground using a grinding machine and passed through a 200-mesh (0.075-mm) sieve. Grinding blade and sieves were carefully sterilized by using 75% alcohol and cleaned with water to avoid cross-contamination between samples. Samples of approximately 20 mg were used to analyze the elemental concentration. The TN and total carbon (TC) contents of the leaves were measured by an elemental analyzer (Vario EL III, Elementar, Hanau, Germany).

### Leaf sampling and measurement

Leaves were sampled from 35 study sites in August 2021, including 21 sites for *G. biloba* and 14 sites for *P. orientalis*. Three healthy individual trees (i.e., three replicates) were selected at each plot. Branches were randomly removed using scissors from four directions (i.e., east, south, west, and north) in the central canopy of the selected tree. Sufficient fresh leaves were collected from the removed 3-year-old branches. There were three replicates of leaf samples for each plot. Leaf samples were kept in ziplock plastic bags and stored with ice bags in a portable cooler box, then transferred to the laboratory. Ten leaves were chosen randomly from each bag to measure the leaf area, leaf relative water content (RWC), and SLA. For the rest of these leaf samples, five leaves were selected randomly and cleaned to remove dust and debris using deionized water. The leaf edges for both species and leaf veins for *P. orientalis* were removed, then cut into strips about 1–2 mm wide using scissors. The leaf strips were kept frozen at -40°C until further processing.

In the laboratory, fresh leaves were first weighed, then the leaf area was measured using a leaf area meter (LI-3000C, Li-Cor, USA), and then leaves were taken to full water saturation for 24 h and weighed again. Finally, the leaves were dried for 72 h at 75°C and weighed again. SLA was calculated as the ratio of the leaf area to the dry leaf weight. RWC was calculated using the following equation:


(1) $$\;\text{RWC }=\;\frac{\left(Fresh\;leaf\;mass\;-\;Dry\;leaf\;mass\right)}{\left(Fully\;water-saturated\;leaf\;mass-Dry\;leaf\;mass\right)}$$

The chlorophyll and carotenoid contents were analyzed using the methods of Li et al. (2018). Fresh leaves (0.1 g) were treated with 10 ml of 95% ethanol solution in the dark for about 48 h at 4°C until completely fading. During the extraction of chlorophyll, the leaves were shaken 6–8 times a day to ensure complete extraction. The chlorophyll (a + b) (Chl a+b) content and carotenoid content were measured by using the specific absorption coefficients (664, 649, and 470 nm) following Lichtenthaler (1987).

The MDA content was analyzed according to the method of Heath and Packer (1968) based on the thiobarbituric acid (TBA) reaction. Fresh leaves (0.1 g) were mixed with 1 ml of 10% trichloroacetic acid (TCA) and ground to homogenate and then centrifuged at 8,000g for 10 min. The supernatant at 100 μl was mixed with 100 μl of 0.6% TBA. The mixture was incubated at 100°C for 60 min and quickly cooled on ice. Then, the solution was centrifuged at 10,000g for 10 min, and the MDA content was measured by using specific absorption coefficients (600, 532, and 450 nm). The MDA values were expressed as the percentage of fresh matter.

The soluble sugar content was determined using the anthrone method proposed by Yemm and Willis (1954). Approximately 0.1 g of fresh leaves were placed into a mortar and then ground to homogenate with 1 ml of distilled water. The solution was bathed for 10 min at 100°C and centrifuged for 10 min at 8,000g. The supernatant at 0.5 ml was diluted with distilled water to a final volume of 10 ml. Then, 40 μl of the solution was mixed with 40 μl distilled water, 20 μl anthrone reagent, and 200 μl of 98% H_2_SO_4_, and the solution was heated at 95°C for 10 min. The solution was cooled to room temperature, and the absorbance was determined spectrophotometrically at 620 nm. The soluble sugar content in the samples was calculated from a standard curve and on a fresh weight basis.

The free proline content was measured according to the method of Bates et al. (1973). Fresh leaves (0.1 g) were extracted in 1 ml of 3% sulfo-salicylic acid. Then, 0.25 ml of the filtrate was mixed with 0.25 ml of acid ninhydrin followed by 0.25 ml of glacial acetic acid. All samples were incubated at 100°C for 60 min and cooled in an ice bath, and 0.5 ml of toluene was added to the solution and mixed vigorously. The chromophore-containing toluene was aspirated, and the absorbance was read at 520 nm on a microplate spectrophotometer (Spectramax 190, Molecular Devices, San Jose, CA, USA). The proline concentration in the samples was determined from a standard curve and calculated on a fresh weight basis.

The element content and carbon stable isotope were analyzed in leaves collected from 15 study sites, the same study sites as the soil samples. The leaf samples were dried at 75°C, ground, and stored in a desiccator until further analysis. The TN and TC contents were determined using the same method as for soil samples, with approximately 7 mg of leaf sample used in the analysis. Relative C stable isotope abundances were determined by combustion in an elemental analyzer (FLASH 2000, Thermo Fisher Scientific, Waltham, MA, USA) operated in continuous-flow mode and connected to an isotope ratio mass spectrometer (Delta V Advantage, Thermo Fisher Scientific, Waltham, MA, USA), with samples of ca. 1.3 mg used in the analysis. The carbon isotope composition (δ^13^C) was used to report the carbon isotope ratio of the sample relative to the standard and was determined by the following equation (2):


(2) $$\delta^{13}\text{C }=\;\left(R_{sample}/R_{standard}-\;1\right)\;\times \;1000\left(‰\right)$$

where *R* is the ratio of the heavy to the light isotopes in the sample or the respective standard. The CO_2_ standard gas calibration, control of reproducibility, and accuracy of the isotope abundance measurements in the leaf samples followed the protocol described by Gebauer and Schulze (1991).

The carbon isotope discrimination, Δ^13^C, was calculated following Farquhar et al. (1982):


(3) $$\Delta^{13}\text{C}=\frac{\left(\delta_{air\;}-\;\delta_{leaf}\right)}{\left(1\;-\;\delta_{leaf}/1000\right)}$$

where *δ_leaf_* is the *δ^13^C* of the leaves and *δ_air_* is the *δ^13^C* of the atmospheric CO_2_ and takes the value of -10‰ ± 0.9‰ according to the results of a site at 10-m altitude in Beijing during the vegetated season (Pang et al., 2016). Given that the leaf conductance to water vapor (*k*) is 1.6 times the conductance to CO_2_, Δ^13^C can be converted to intrinsic water-use efficiency (iWUE) as follows (Ma et al., 2021):


(4) $$\text{iWUE }=\;\frac{c_{a}}{k}\cdot \frac{b-\Delta -f^{\prime }\frac{{\text{Γ}}^{*}}{c_{a}}}{b-a_{s}+\frac{g_{sc}}{g_{m}}\cdot (b-a_{m})}$$

where *c_a_* is the CO_2_ mole fraction in the atmosphere; *b* (29‰) and *f* (11‰) represent the fractionations due to Rubisco carboxylation and photorespiration, respectively; *a_b_* = 1 + *b*, *a_f_* = 1 + *f*, and *f’* = *fa_b_*/*a_f_*. Γ* (35) is the CO_2_ compensation point in the absence of mitochondrial respiration; *a_s_* (4.4‰) is the fractionation during CO_2_ diffusion through the stomata; *g_sc_*/*g_m_* (0.8) was the ratio between the stomatal conductance to CO_2_ (*g_sc_*) and the mesophyll conductance (*g_m_*). *a_m_* (1.8‰) is the fractionation associated with CO_2_ dissolution and diffusions in mesophyll.

### Statistical analyses

The effects of the pavement and land use types on the environmental factors, tree growth, and morphological and physiological traits were tested by a linear mixed-effects model (LMM) using the “lmer” function in LMER4 package (Bates et al., 2015). The pavement type and land use type were modeled as fixed factors and the site as a random effect to account for the nested sampling design. Tukey's honest significant difference (HSD) test was performed using the R package “emmeans” to test differences between the paved land and the unpaved land. Pearson’s correlation coefficients were calculated between the tree growth and foliar traits to investigate the effect of the leaf physiological and morphological traits, δ^13^C, and leaf and soil nutrient content on the tree growth.

To determine whether the leaf morphological or physiological traits had a stronger link to the tree growth in *G. biloba* and *P. orientalis*, we performed structural equation models (SEMs) using the R packages “piecewiseSEM” (Lefcheck, 2016). Without an established framework for the leaf trait–growth relationships, we initially excluded these from the model framework. Fisher’s C statistic was used as the goodness-of-fit, and the final model was considered to have an adequate overall fit to the observed data when the model had a nonsignificant C value (*P* > 0.05) (Shipley, 2009).

The data analysis was conducted using the R language (R, version 4.0, http://www.R-project.org).

## Results

### Environmental factors

**Figure 3:**
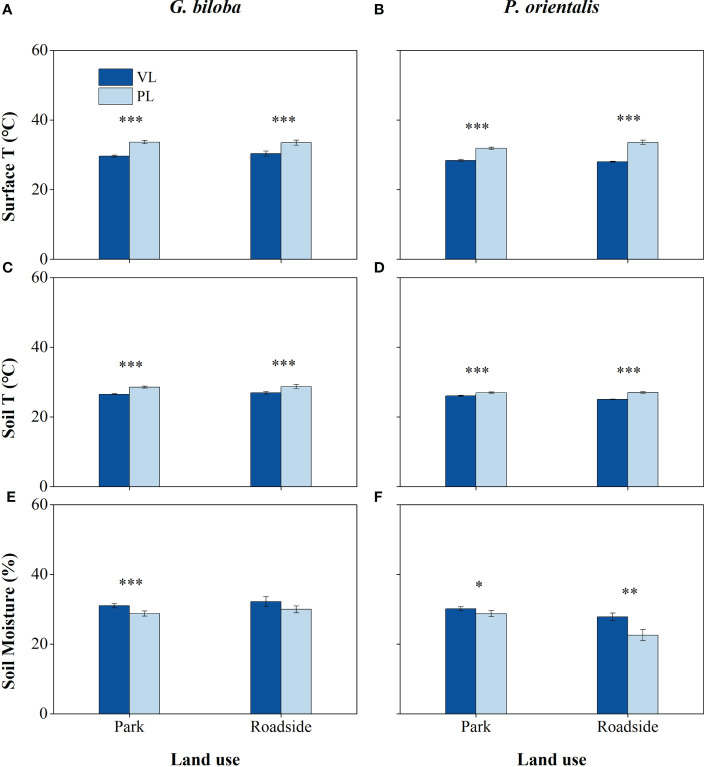
The surface temperature **(A, B)**, soil temperature **(C, D)**, and soil moisture **(E, F)** of the vegetated land (VL) and the paved land (PL) at the park and roadside for *Ginkgo biloba* and *Platanus orientalis*. Error bars indicate the standard error of the mean. *, **, *** indicate statistically significant differences between VL and PL at *P*< 0.05, *P*< 0.01, and *P*< 0.001, respectively.

**Figure 4:**
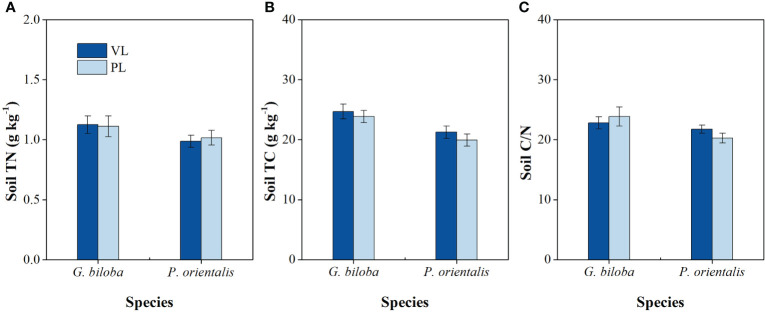
The differences in the soil total nitrogen (TN) **(A)**, soil total carbon (TC) **(B)**, and soil C/N ratio **(C)** between the vegetated land (VL) and the paved land (PL) at the park for *Ginkgo biloba* and *Platanus orientalis*. Error bars indicate the standard error of the mean.

There were significant differences in the surface temperature, soil temperature, and moisture between the paved land and the vegetated land in both parks and roadsides for both species except for the soil moisture of *G. biloba* in roadsides (**Figure 3**). Compared with the vegetated land, the surface temperatures in the paved land were 4.02°C and 3.15°C higher in parks and roadsides for *G. biloba*, respectively (*P*< 0.001), and 3.47°C and 5.60°C higher in parks and roadsides for *P. orientalis*, respectively (*P*< 0.001); the soil temperatures in the paved land were 2.00°C and 1.80°C higher at parks and roadsides for *G. biloba*, respectively (*P*< 0.001), and 0.92°C and 1.94°C higher at parks and roadsides for *P. orientalis*, respectively (*P*< 0.001); and the soil moistures in the paved land were 2.24% lower (*P*< 0.001) in parks for *G. biloba* and 1.45% and 5.27% lower in parks and roadsides for *P. orientalis*, respectively (*P*< 0.05). No significant differences in the soil TN, TC, and C/N ratios were found between the paved land and the vegetated land in parks for both species (**Figures 4A–C**).

### Tree growth

Relative to the vegetated land (**Figure 2**), the mean DBH increment, height increment, and LAD in the paved land were significantly decreased by 44.5%, 31.9%, and 18.9% for *G. biloba* and by 31.7%, 60.1%, and 18.7% for *P. orientalis*, respectively; the crown increment of *G. biloba* in the paved land significantly decreased by 44.9% (*P*< 0.05).

The effects of the pavement type on the tree growth varied with the land use type (**Figure 2**). Relative to the vegetated land, the reductions in the DBH increment in the paved land were significantly (*P*< 0.05) higher at the park than at the roadside for *G. biloba*, while the reduction in the LAD was significantly (*P*< 0.001) lower at the park than at the roadside for *P. orientalis*.

### Leaf morphology and physiology

**Figure 5:**
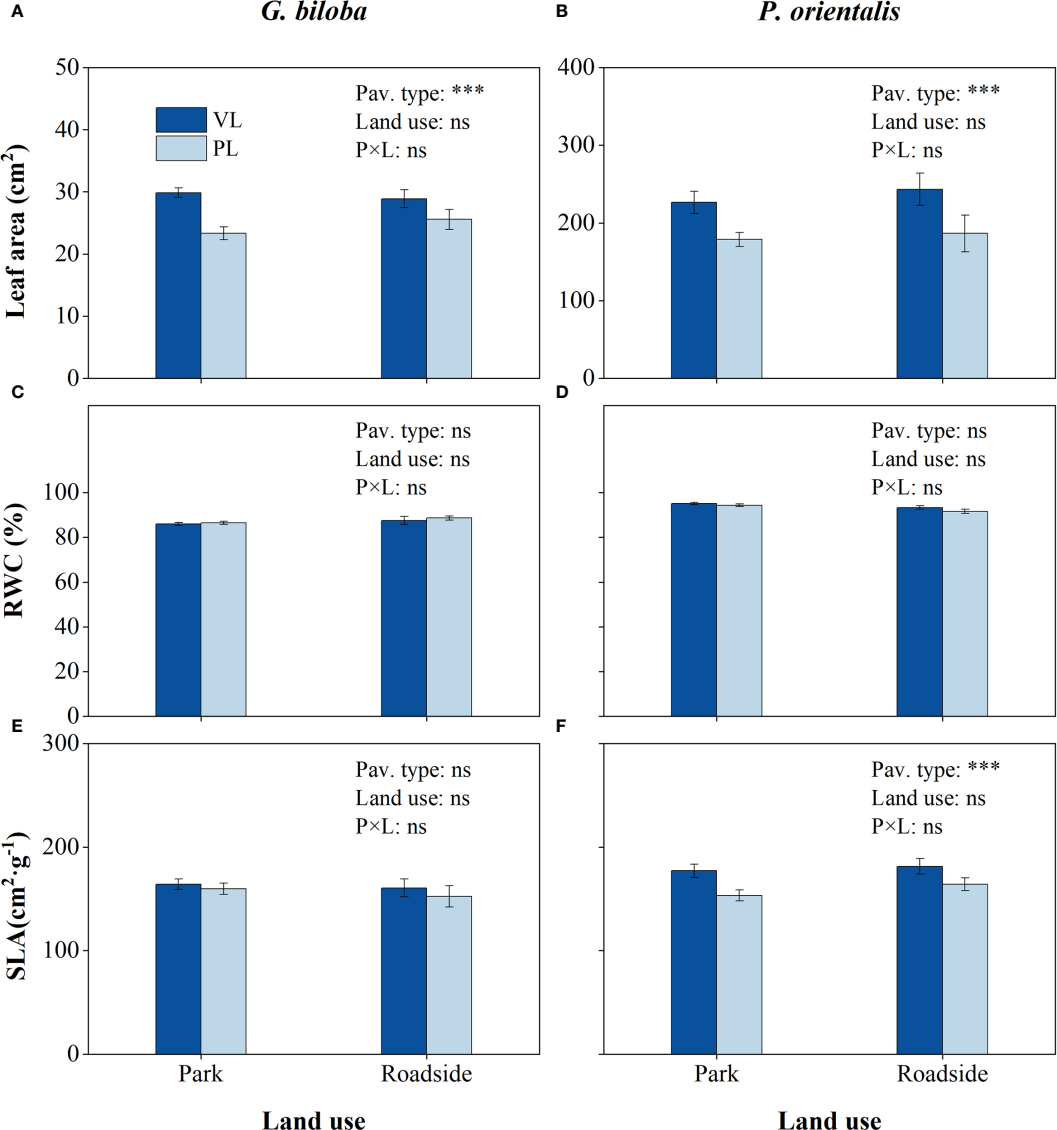
Results from the linear mixed-effects model (LMM) testing for the differences in leaf area **(A, B)**, relative water content (RWC) **(C,D)**, and specific leaf area (SLA) **(E, F)** vs. the pavement type (VL, vegetated land; PL, paved land), land use (park and roadside), and their interactions. ***, *P*< 0.001; ns, not significant. Data represent mean ± SE.

Compared with the vegetated land (**Figure 5**), the mean leaf area in the paved land significantly (*P*< 0.001) decreased by 20.0% and 21.6% for *G. biloba* and *P. orientalis*, respectively; the mean SLA in the paved land significantly (*P*< 0.01) decreased by 12.6% for *P. orientalis*.

**Figure 6:**
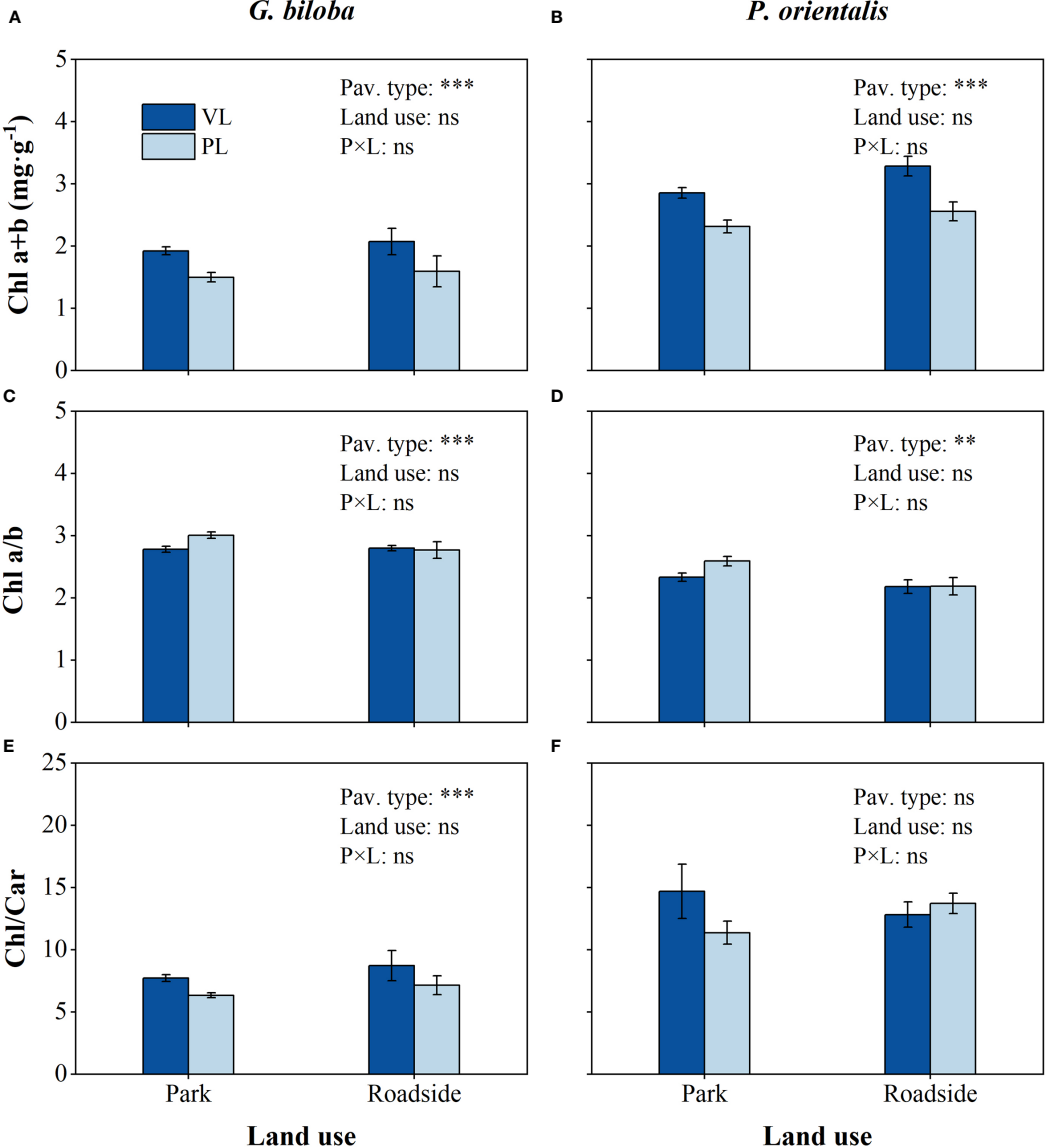
Results from the linear mixed-effects model (LMM) testing for the differences in the content of Chl a+b **(A, B)**, Chl a/b **(C, D)**, and Chl/Car **(E, F)** vs. the pavement type (VL, vegetated land; PL, paved land), land use (park and roadside), and their interactions. **, *P*< 0.01; ***, *P*< 0.001; ns, not significant. Data represent mean ± SE.

Relative to the vegetated land (**Figure 6**), the mean Chl a+b content in the paved land significantly decreased by 22.2% and 19.6% for *G. biloba* and *P. orientalis*, respectively (*P*< 0.001), and the mean Chl/Car by 17.8% for *G. biloba*; the mean Chl a/b in the paved land significantly increased by 6.3% and 8.9% for *G. biloba* and *P. orientalis*, respectively.

**Figure 7:**
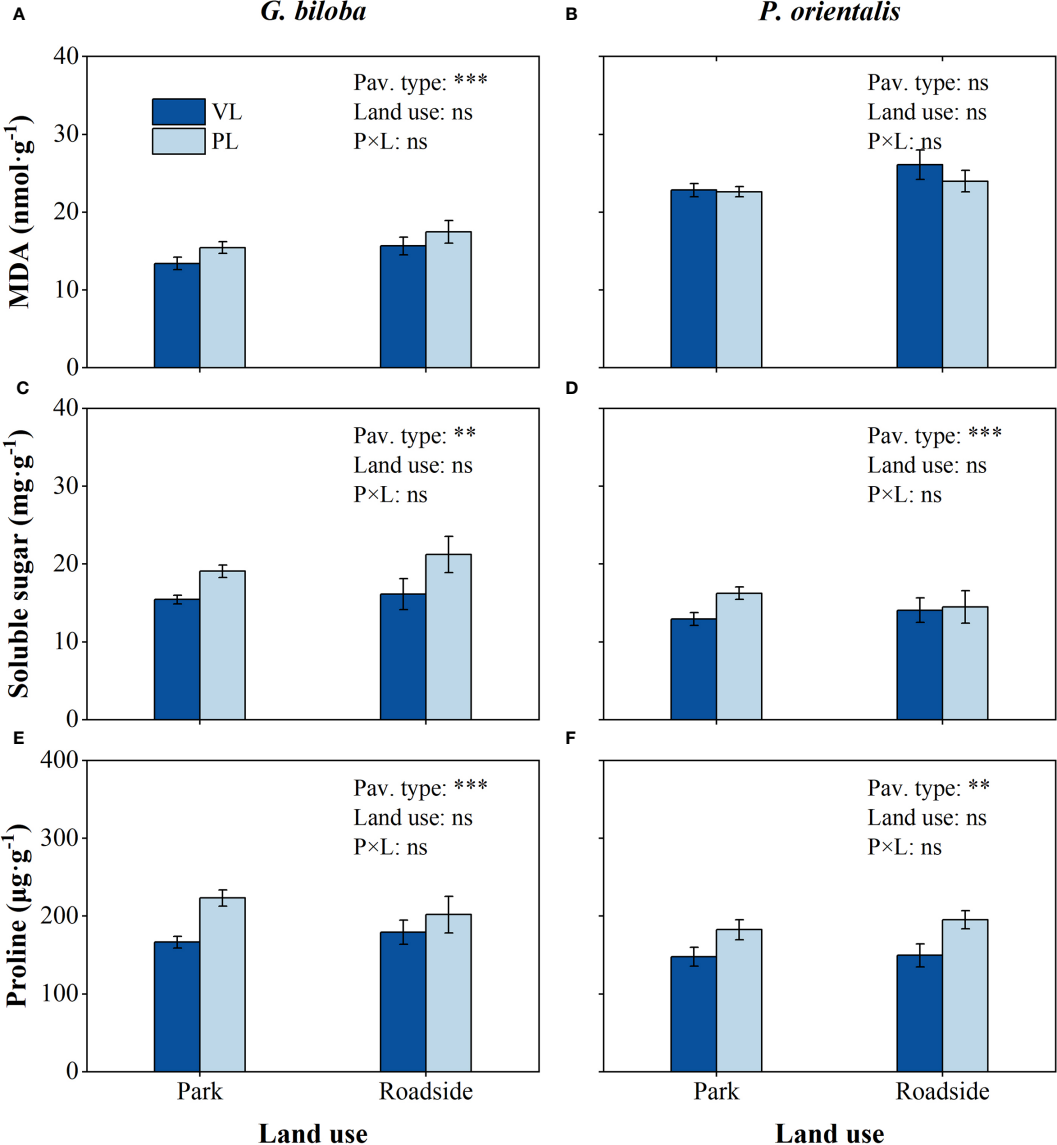
Results from the linear mixed-effects model (LMM) testing for the differences in the content of Malondialdehyde (MDA) **(A, B)**, soluble sugar **(C, D)**, and proline **(E, F)** vs. the pavement type (VL, vegetated land; PL, paved land), land use (park and roadside), and their interactions. **, *P*< 0.01; ***, *P*< 0.001; ns, not significant. Data represent mean ± SE.

Relative to the vegetated land (**Figure 7**), the mean soluble sugar content in the paved land significantly (*P*< 0.01) increased by 25.2% and 20.5% and proline content by 29.7% and 25.1% for *G. biloba* and *P. orientalis*, respectively; the mean MDA content in the paved land significantly increased by 14.4% for *G. biloba*.

### Leaf total nitrogen and total carbon content and carbon isotope composition (δ^13^C)

**Figure 8:**
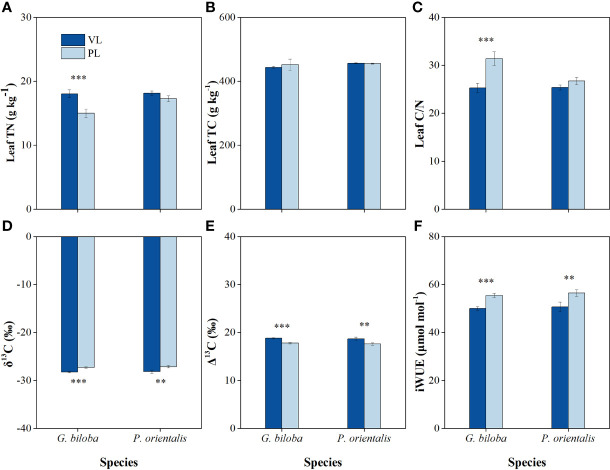
The differences in the leaf total nitrogen (TN) **(A)**, leaf total carbon (TC) **(B)**, leaf C/N ratio **(C)**, carbon isotope composition (δ^13^C) value **(D)**, carbon isotope discrimination (Δ^13^C) value **(E)**, and intrinsic water-use efficiency (iWUE) **(F)** between the vegetated land (VL) and the paved land (PL) at the park for *G biloba* and *P. orientalis*. Error bars indicate the standard error of the mean. ** and *** indicate statistically significant differences between VL and PL at *P*< 0.01 and *P*< 0.001, respectively.

Compared with the vegetated land, the leaf TN and C/N ratio in the paved land significantly decreased by 16.8% and 24.1% for *G. biloba*, respectively (**Figures 8A, C**); the δ^13^C values in the paved land were 0.96‰ and 1.01‰ higher for *G. biloba* and *P. orientalis*, respectively; the iWUE values in the paved land were 10.9% and 11.3% higher for *G. biloba* and *P. orientalis*, respectively; and the Δ^13^C values in the paved land were 1.01‰ and 1.06‰ lower for *G. biloba* and *P. orientalis*, respectively (**Figures 8D–F**).

### Correlation between the tree growth and the foliar traits

**Figure 9:**
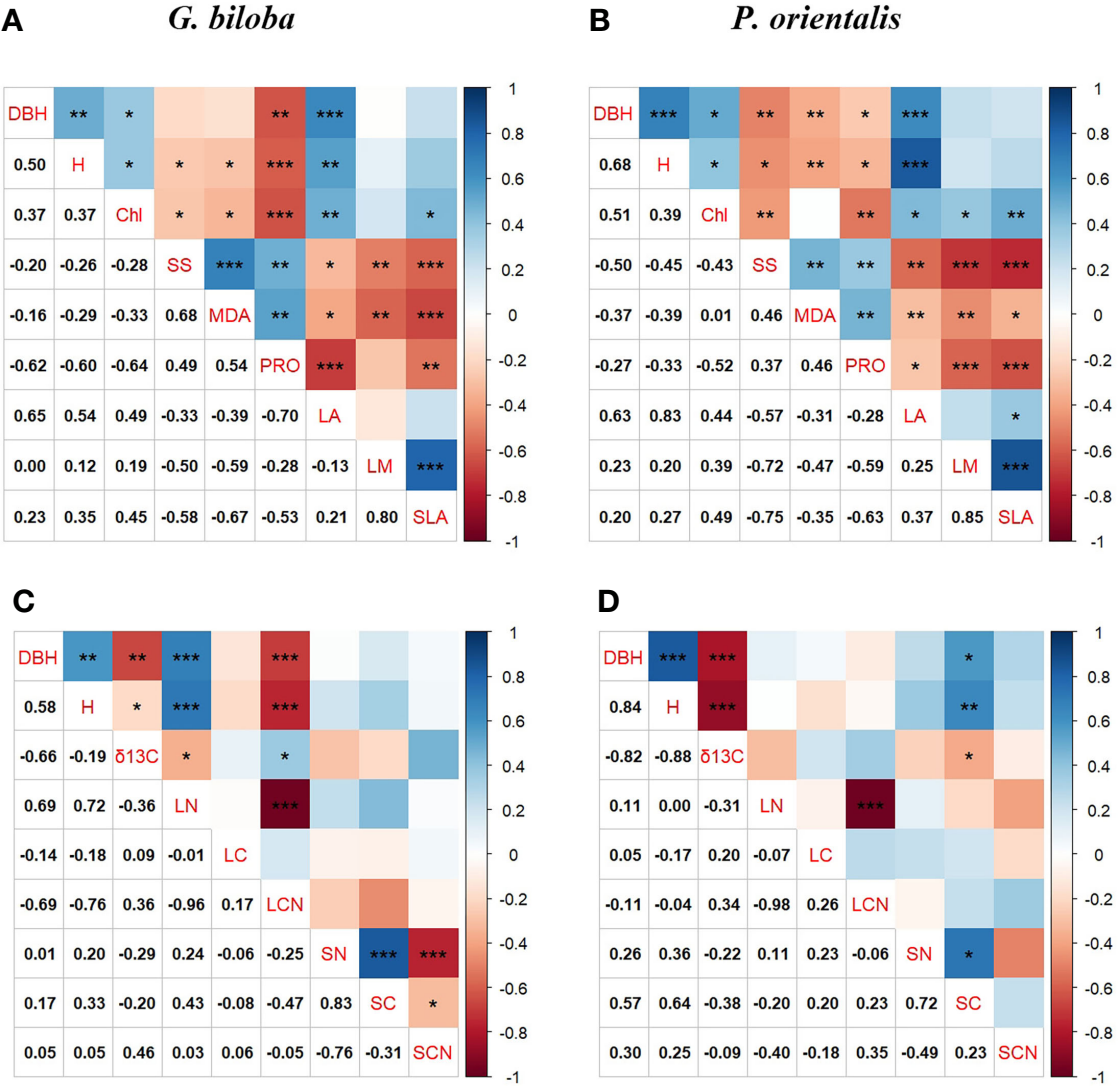
Pearson’s correlations among the tree growth, leaf physiology, and leaf morphology for *G biloba* **(A)** and *P. orientalis* **(B)**, respectively, and among the tree growth, carbon isotope composition (δ^13^C), and leaf and soil nutrient content for *G biloba* **(C)** and *P. orientalis* **(D)**, respectively. Asterisks represent statistical significance (*, *P*< 0.05; **, *P*< 0.01; ***, *P*< 0.001).

At the parks, the tree growth was significantly related to the leaf area but not the leaf water content and SLA for *G. biloba* and *P. orientalis* (*P*< 0.05; **Figures 9A, B**). As for the leaf physiology, the tree growth was significantly positively associated with the chlorophyll but negatively with the soluble sugar, MDA, and proline for both species. The tree growth and δ^13^C also showed a significantly positive correlation for both species (*P*< 0.05; **Figures 9C, D**). In addition, the leaf TN was significantly positively correlated with the tree growth for *G. biloba*.

### Tree growth response to the pavement in relation to the leaf area and proline

**Figure 10:**
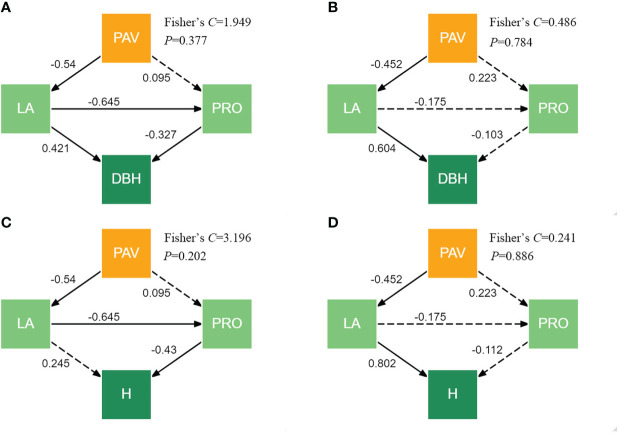
Piecewise SEMs showing the direct and indirect effects of the pavement (PAV), leaf area (LA), and proline (PRO) content on the DBH and height (H) for *G. biloba* **(A, C)** and *P. orientalis* **(B, D)**. Solid arrows represent significant effects (*P*< 0.05), and the dashed arrows indicate nonsignificant effects (*P* > 0.05).

Piecewise SEMs provide evidence that the response of the tree growth to the pavement can be negatively related to the leaf area indirectly through the proline content for *G. biloba*. Although there were no significantly direct relations of the pavement to the proline content in *G. biloba* (**Figure 10**), the proline content can be indirectly related to the pavement through the leaf area (*P*< 0.05; **Figures 10A, C**). The proline content was related to the DBH and height of *G. biloba* (**Figures 9A, B**). Therefore, the DBH of *G. biloba* can be inhibited by the leaf area directly and also indirectly through the proline content. The height of *G. biloba* was only indirectly affected by the leaf area through the proline content.

## Discussion

### Stresses of the paved land on the tree growth

Trees growing on the paved land were strongly inhibited. This study showed that the DBH increment and the height increment significantly declined in the paved land, as widely observed in previous studies with different pavements (Sanders and Grabosky, 2014; Sand et al., 2018).

A higher LAD would block more amount of solar radiation and has a higher cooling effect on the surface temperature (Gillner et al., 2015). In addition, all materials of the paved surface in urban settings usually have a low albedo and thermal capacity, absorbing a large amount of solar radiation, and no evaporation can occur (Krayenhoff and Voogt, 2010; Fini et al., 2017), then the paved land heats up considerably. In conclusion, the paved land has a higher surface and soil temperature as shown in this study (**Figure 3**). The soil water balance mainly depends on the water input, soil evaporation, and plant transpiration (Daly and Porporato, 2005). The paved surface has low permeability (Chen et al., 2011; Pataki et al., 2011; Savi et al., 2015), preventing or delaying rainwater infiltration into the soil. Therefore, the soil moisture under the paved land was lower than that under the vegetated land.

For most temperate tree species, the optimal temperature range for the root is 10°C–25°C (Pregitzer et al., 2000). The root growth and leaf conductance would be decreased when the soil temperature is greater than 30°C (Graves, 1994). A high soil temperature can reduce the soil and plant hydraulic conductance and water uptake (Kramer and Boyer, 1995) by increasing the water viscosity and membrane permeability and also by reducing the new fine root production (Rahman et al., 2014). Therefore, the pavement-induced high temperature might contribute to the reductions in tree DBH and height increment for *G. biloba* and *P. orientalis* on the paved land. In the continental climate with unevenly seasonal distribution in rainfall, such as Beijing, the soil moisture is one of the important factors limiting the tree growth. Under the paved land, rainfall is intercepted, and the soil moisture might even worsen, as shown in this study (**Figure 3**). The pavement-induced low soil moisture might be another cause of reduction in the tree growth.

In addition, urban areas are characterized by huge microclimatic heterogeneity depending on the structure of the sites (Moser et al., 2017). Compared with the vegetated land, the paved land has been shown to have high levels of solar radiation (Kjelgren and Clark, 1992), air temperature, and vapor pressure deficit (Kirschbaum, 2004) and further enhance the evaporative demand, then exacerbate drought stress to trees in the paved land (Moser et al., 2016). So, in a future study, it would better to monitor more factors for each tree.

Air pollution could have a stronger influence than the climate on the tree growth (Locosselli et al., 2019). Matsumoto et al. (2022) found that *Rhododendron pulchrum*, *Rhaphiolepis*, and *Prunus yedoensis* all had a higher restriction of maximum photosynthesis. Therefore, the combined effects of the pavement and air pollution require further study in the future.

### Alterations of the leaf morphology and physiology under the paved land

The leaf is the critical component for the tree to utilize light energy and evaporate water that are essential to drive biological processes. However, the leaf is easily altered by changes in the environment. The stresses of the paved land would alter the leaf morphology and physiology. This study showed significant reductions in the leaf area, chlorophyll, and N contents and increases in the MDA and δ^13^C for *G. biloba* and/or *P. orientalis*.

Under drought stress brought by the paved land, the leaf area of both species and SLA of *P. orientalis* become smaller in this study. It is generally thought that smaller leaves can shed heat more quickly to reduce the evaporative cooling in drier climates (McDonald et al., 2003). Plants with lower SLA tend to devote most of their energy and nutrients to building defensive structures or increasing leaf tissue density to prevent excessive dehydration (Garnier et al., 2001). In contrast, Zhang et al. (2022) reported a lower SLA but not leaf size in *Cinnamomum camphora* under an impervious pavement. Therefore, the responses of the leaf morphology to the pavement can be species specific.

As a component of the photosynthetic machinery, chlorophyll absorbs light energy and is involved in energy transfer in the course of photosynthesis (Von Wettstein et al., 1995; Blankenship, 2002). In this study, the leaves in the paved land have a significantly declined chlorophyll content. This can be caused by the reduced biochemical activities of the chlorophyll biosynthetic enzymes under drought stress (Dalal and Tripathy, 2012). Meanwhile, plants would increase the mass fraction of the non-photosynthetic (vascular and sclerenchyma) tissues under drought stress, which allows optimization of water use (Ivanova et al., 2018). The reduced chlorophyll content on the paved land will limit photosynthesis and the tree growth.

A significant decline in the leaf TN content under the paved land occurred for *G. biloba* in this study (**Figure 4**). However, there is no significant difference in the soil TN content. Therefore, the declined leaf TN content can be caused by the declined uptake of plant nutrients due to the reduced transpiration under drought stress. As one of the main macroelements needed for plant growth, nitrogen in plants is an elementary component of the chlorophyll nucleic acids, multiple coenzymes, vitamins, and plant hormones (Hammad and Ali, 2014). Therefore, a low leaf TN could reduce photosynthesis (Evans, 1989b) and then the growth of *G. biloba*.

As the final product of plasma membrane peroxidation, the molality concentration of MDA can reflect the degree of plant damage (Mittler, 2002; Zandalinas et al., 2016). In this study, only *G. biloba* showed a significant increase in MDA under the paved land, indicating a higher degree of damage to cell membranes for *G. biloba*.

### Adaptation of the trees to the paved land

Plants could adapt to an unfavorable environment by several biochemical or biophysical processes, such as the regulation of osmotic balance and the ratio of photosynthetic pigments, and enhancement of the water-use efficiency.

Under drought stress, plants will regulate the osmotic balance to decrease the osmotic potential and maintain the turgor of the mesophyll (Sevanto et al., 2014) by accumulating osmotic adjustment substances such as soluble sugar and proline (Ashraf and Foolad, 2007; Szabados and Savoure, 2010; Lamke and Baurle, 2017; Bechtold and Field, 2018). In this study, the leaf proline and soluble sugar were significantly increased under the paved land in *G. biloba* and *P. orientalis* leaves, which is consistent with the reports on *Camellia oleifera* under drought stress (He et al., 2020), indicating that there is an adaptive mechanism for trees to regulate osmotic substances under the paved land. According to the optimal nitrogen partitioning among the photosynthetic components (Evans, 1989a; Hikosaka and Terashima, 1995), the Chl a/b ratio increases in response to N limitation (Kitajima and Hogan, 2003). Controlling the Chl a/b ratio was also one way to adapt the photosynthetic function to high-light and arid environments (Björkman, 1981). Therefore, the increased Chl a/b ratio could be an adaptation to lower soil moisture under the paved land for both species and also an adaptation to lower TN for *G. biloba*. The de-epoxidized carotenoid zeaxanthin plays additional important roles as a thylakoid stabilizer and as an antioxidant upon desiccation (Havaux, 1998; Fernandez-Marin et al., 2013). Therefore, the lower Chl/Car is also a measure of chloroplast protection (Fernandez-Marin et al., 2020).

Under the paved land, leaf δ^13^C abundance increased (**Figure 8**). Plants discriminate against ^13^C during photosynthetic CO_2_ fixation (Duranceau et al., 1999). Enzymes in the photosynthetic pathway would discriminate against the heavier molecule, ^13^CO_2_, and preferentially use the lighter molecule, ^12^CO_2_ (Mullaney et al., 2015a). While under drought stress, a greater proportion of ^13^CO_2_ is fixed in leaves owing to the reduced internal ^12^CO_2_ concentrations in leaves caused by stomatal closure (Farquhar et al., 1989; Handley et al., 1997). The δ^13^C enrichment under the paved land could reduce photosynthesis due to the reducing gas exchange and then inhibited the growth. Meanwhile, as one strategy to adapt to drought stress, the plant must save water use for production, i.e., enhance water-use efficiency. In this study, the time-integrated measurements of iWUE are inferred from the ^13^C stable isotopic composition (δ^13^C) of plant material (Farquhar et al., 1989). Under the paved land, the iWUE increased significantly for both *G. biloba* and *P. orientalis*, implying that trees have adapted to the paved land by the tradeoff between carbon use and water loss for trees in the pavement. Kagotani et al. (2013) also found that the long-term leaf WUE of *Prunus yedoensis* is higher at the sites with a high air temperature and low soil water content due to a smaller leaf Δ^13^C in Kyoto city.

### Compounding effects of the leaf morphological and physiological traits

In this study, there were significant correlations between the leaf morphological and physiological traits (**Figure 9**), indicating that trees growing on the paved land have altered simultaneously in leaf morphological and physiological properties.

Leaf photosynthesis is subjected to both stomatal limitations and non-stomatal limitations (NSLs) (Buckley and Diaz-Espejo, 2015). Generally, stomatal limitations and NSLs to photosynthesis operate concurrently and are coordinated with each other (Flexas et al., 2008; Gago et al., 2016). Under the paved land, a small leaf area incurs less stomata for taking up CO_2_ into carbohydrates. The simultaneous changes in the leaf morphology and physiology will reduce the photosynthetic rate per area. In addition, the LAD under the paved land was significantly lower than under the vegetated land. Then, the photosynthetic production as well as the growth of an individual tree was reduced. The close relationships between leaf traits and tree growth (**Figure 9**) showed that each trait of either leaf morphology or physiology influences the tree growth independently. However, these leaf traits might influence the tree growth dependently. As shown in **Figures 10A, C**, the leaf area reduction under the paved land influences the DBH of *G. biloba* both directly and indirectly through the proline content and the height of *G. biloba* not directly but through the proline content. This implies the importance of the confounding effects of the leaf morphology and physiology in understanding the mechanism underlying the tree response to environmental stress.

### Species difference in the tolerance to the paved land

This study confirmed that both tree species investigated are significantly impacted by the paved land in growth and leaf morphology and physiology; however, there was still a difference between *G. biloba* and *P. orientalis* in the tolerance to the paved land. The leaf MDA content increased under the paved land significantly for *G. biloba* but not for *P. orientalis*, indicating that the ability to preserve the membrane stability is weaker for *G. biloba* than for *P. orientalis*. The leaf TN content was reduced by the paved land significantly for *G. biloba* but not for *P. orientalis*. All of these suggest that *G. biloba* might be more vulnerable to the stress under the paved land than *P. orientalis*. While only *P. orientalis* had a significant decrease in the SLA under the paved land. All of these may be caused by the differences in the anatomy of the leaf venation structure and the stem xylem structure. As a palmately veined species, *P. orientalis* has main veins but not *G. biloba*, with parallel veins in dichotomy, and a higher vein length per unit area (VLA) than *G. biloba* (Sack and Scoffoni, 2013). Under drought stress, the main vein density and thick-walled conduits of *P. orientalis* are both increased, implying increased construction costs per unit surface area and then lower SLA. *P. orientalis* is a diffuse-porous species while *G. biloba* is a non-porous species. Diffuse-porous species usually had a higher water transport efficiency than non-porous species under drought stress (Dai et al., 2020). Therefore, there were different adaptation mechanisms to the pavement in the SLA, leaf TN content, and MDA for *G. biloba* and *P. orientalis*.

Because of its importance to cultural and esthetic values in China, *G. biloba* has become one of the most popular tree species in urban greening in China ranging from subtropical to temperate climatic zones. Considering the low tolerance to the paved land, *G. biloba* is not recommended for planting on the paved land except when a good irrigation system is guaranteed.

## Conclusions

Trees have been planted widely on the paved land for offsetting the limitation of land and providing more ecosystem services in the urban context. The high soil temperature and low soil moisture have exerted significant stress on the tree growth, which was manifested in the field by decreased DBH and height increments, leaf area, and leaf chlorophyll content but increased MDA, as compared with the vegetated land. However, trees could adapt to the paved land stress by regulating the osmotic balance through increasing the leaf proline and soluble sugar contents and increasing water-use efficiency through decreasing the leaf area and stomatal conductance. The reduction in tree growth on the paved land is related to the changes in the leaf morphology and physiology. Compared with *G. biloba*, *P. orientalis* showed different adaptations to the pavement-induced stresses in the SLA, leaf TN content, and MDA, which imply that they responded by different pathways. To mitigate high-temperature and low-moisture stresses on trees growing on paved lands, silvicultural measures such as irrigation should be conducted in time.

## Data availability statement

The raw data supporting the conclusions of this article will be made available by the authors, without undue reservation.

## Ethics statement

Written informed consent was obtained from the individual(s) for the publication of any potentially identifiable images or data included in this article.

## Author contributions

BC and XKW designed the study. BC, YS and DZ conducted the field survey and laboratory analyses. BC, XMW and CG analyzed the data. BC and XMW wrote the manuscript. XKW and ZO reviewed and edited the manuscript. All authors contributed to the article and approved the submitted version.

## Acknowledgment

We especially thank Chao Wang, Yang Yao, Zhen Li and Shuai Zhang for the field work. Special appreciations are given to the reviewers for their constructive suggestions on revision. This work was supported by grants from the National Natural Science Foundation of China (No. 41571053 and No. 71533005).

## Conflict of interest

The authors declare that the research was conducted in the absence of any commercial or financial relationships that could be construed as a potential conflict of interest.

The handling editor declared a shared affiliation with the authors BC, CG, DZ, ZO and XW at the time of the review.

## Publisher’s note

All claims expressed in this article are solely those of the authors and do not necessarily represent those of their affiliated organizations, or those of the publisher, the editors and the reviewers. Any product that may be evaluated in this article, or claim that may be made by its manufacturer, is not guaranteed or endorsed by the publisher.
